# Ginseng-Sanqi-Chuanxiong (GSC) Extracts Ameliorate Diabetes-Induced Endothelial Cell Senescence through Regulating Mitophagy via the AMPK Pathway

**DOI:** 10.1155/2020/7151946

**Published:** 2020-09-07

**Authors:** Xue Wang, Jia-Qi Zhang, Cheng-Kui Xiu, Jing Yang, Jing-Yi Fang, Yan Lei

**Affiliations:** ^1^Beijing Key Laboratory of Research of Chinese Medicine on Preventional and Treatment for Major Diseases, Experimental Research Center, China Academy of Chinese Medical Sciences, 100700 Beijing, China; ^2^Xiyuan Hospital, China Academy of Chinese Medical Sciences, 100700 Beijing, China; ^3^Chinese Medicine Research Institute of Guangdong Pharmaceutical University, Guangdong Research Center for Integrative Medicine in Metabolic Diseases, 510006 Guangzhou, Guangdong, China

## Abstract

Vascular endothelial senescence induced by high glucose and palmitate (HG/PA) contributes to endothelial dysfunction, which leads to diabetic cardiovascular complications. Reduction of endothelial senescence may attenuate these pathogenic processes. This study is aimed at determining whether Ginseng-Sanqi-Chuanxiong (GSC) extracts, traditional Chinese medicine, can ameliorate human aortic endothelial cell (HAEC) senescence under HG/PA-stressed conditions and further explore the underlying mechanism. We found that GSC extracts significantly increased antisenescent activity by reducing the HG/PA-induced mitochondrial ROS (mtROS) levels in senescent HAECs. GSC extracts also induced cellular mitophagy formation, which mediated the effect of GSC extracts on mtROS reduction. Apart from this, the data showed that GSC extracts stimulated mitophagy via the AMPK pathway, and upon inhibition of AMPK by pharmacological and genetic inhibitors, GSC extract-mediated mitophagy was abolished which further led to reverse the antisenescence effect. Taken together, these data suggest that GSC extracts prevent HG/PA-induced endothelial senescence and mtROS production by mitophagy regulation via the AMPK pathway. Thus, the induction of mitophagy by GSC extracts may provide a novel therapeutic candidate for cardiovascular protection in metabolic syndrome.

## 1. Introduction

Diabetes mellitus is a metabolic disease characterized by elevated levels of glucose and free fatty acids, and it has become a major threat to human health due to its severe cardiovascular complications [1]. Accumulating evidence has shown that chronic exposure of the vascular endothelium to hyperglycemia (HG), hyperlipidemia (HL), or both leads to endothelial senescence preceding and promotes vascular inflammation and atherosclerosis resulting in the development of diabetic complications [2]. Cellular senescence is a phenotype of permanent growth arrest that occurs in response to various cellular stress and damage, which is accompanied by molecular reprogramming and secretion of a set of inflammatory cytokines and chemokines, growth factors, and proteases, maintaining the senescence arrest via self-amplifying positive feedback loops and inducing senescence in healthy cells in a paracrine fashion [3–6]. Overproduction of mitochondrial reactive oxygen species (mtROS) has been shown to play an important role in cellular senescence [7]. HG/HL accelerates oxidative damage to mitochondria by increasing production of mtROS, in turn, triggering organelles to produce more mtROS and potentiating the development of cellular senescence and diabetic complications [8]. Therefore, identification agents or mechanisms for protecting endothelial cells from metabolic stress-induced cellular senescence have become a focus of attention in the treatment of diabetes mellitus.

Mitophagy is a form of selective autophagy, which can mark damaged mitochondria for autophagy recognition and degradation, playing a key role in mitochondrial and cellular homoeostasis [9]. The damaged mitochondria would be the main source of mtROS production if they cannot be cleared in time. Mitophagy is activated by depolarizing the mitochondrial membrane and stabilizes the phosphatase and tensin homolog-induced putative kinase 1 (PINK1) on the mitochondrial outer membrane, which then recruits E3 ubiquitin ligase Parkin from the cytoplasm and amplifies the initiation signals of mitophagy [10]. Impaired mitophagy has been reported to relate to premature aging and age-associated disorders [11–13].

Adenosine monophosphate-activated protein kinase (AMPK), as an energy sensor and regulator, plays a fundamental role in cellular and organismal energy metabolism [14]. The mammalian target of rapamycin (mTOR) is a critical downstream target for AMPK, involving multiple cellular processes such as the cell cycle, cell growth, and autophagy [15]. AMPK can directly phosphorylate the regulatory component of mTORC1 (Raptor protein) or the inhibitor of the mTOR complex (tuberous sclerosis protein 2 (TSC2)) to inhibit mTOR activity. Recent studies have found that the AMPK signaling pathway is related to the process of mitophagy [16, 17]. In different cell lines, mitochondrial damage induced by carbonyl cyanide 3-chlorophenylhydrazone (CCCP) can cause physical binding of AMPK to autophagy-related genes and induce the VPS34-ATG16 complex to be recruited to damaged mitochondria [18].

The traditional Chinese medicine Ginseng-Sanqi-Chuanxiong (GSC) is composed of Panax ginseng C. A. Mey., Panax notoginseng (Burk.) F. H. Chen, and Ligusticum chuanxiong Hort. at a ratio of 2 : 3 : 4, which has been applied for cardiovascular and diabetic treatment. Recently, pharmacological studies have shown that the active ingredients of these three drugs have antiaging effects [19–21]. Our previous studies showed that GSC extracts can alleviate vascular aging in rat and mouse models [22, 23]. Moreover, GSC extracts can ameliorate vascular endothelial and smooth muscle cell senescence by regulating the renin-angiotensin-aldosterone system, NO synthesis, and oxidative stress [24, 25]. However, the cytoprotective and antisenescence role of GSC extracts by mitophagy regulation via the AMPK pathway in HG/HL-induced endothelial cell senescence still remains unclarified. In this study, we explored the possible pharmacological effects of GSC extracts in the inhibition of cellular senescence by regulating the AMPK pathway-mediated mitophagy in HAECs under HG/HL-stressed conditions, thus providing a new perspective for the prevention and treatment of diabetic cardiovascular complications.

## 2. Materials and Methods

### 2.1. Preparation of GSC Extracts

**Figure 1:**
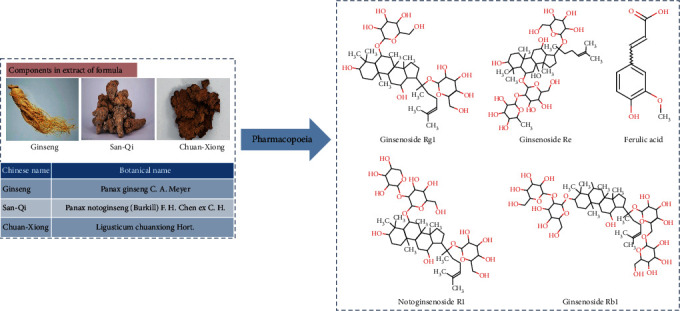
The components of the GSC formula.

GSC consists of three herbs, and the mixed proportion of respective herbs in GSC is illustrated in Figure 1. Ethanol extracts of GSC were provided by Beijing YinKeRuiSi Medical Technology Co. Ltd. Raw herbs were prepared into dry powder through ethanol extraction, concentration, and vacuum decompression. The dry powder was concentrated with a final concentration of 4.286 g dried crude herb per g. According to the pharmacopoeia, the five chief components (ferulic acid, notoginsenoside R1, ginsenoside Rg1, ginsenoside Re, and ginsenoside Rb1) of GSC extracts were analyzed. Chromatographic analysis showed that the concentrations of ferulic acid, notoginsenoside R1, ginsenoside Rg1, ginsenoside Re, and ginsenoside Rb1 were 1.00 mg/g, 8.45 mg/g, 55.84 mg/g, 6.47 mg/g, and 44.57 mg/g, respectively (as shown in the supplement file (available here)). GSC extracts were dissolved in DPBS, then filtered and stored in -80°C until used.

### 2.2. Cells and Treatment

Human aortic endothelial cells (HAECs, cat. no. 6100) were purchased from ScienCell Research Laboratories (USA) and cultured in endothelial cell medium (ECM, cat. no. 1001) supplemented with 5% FBS and penicillin/streptomycin antibiotic solution; endothelial cells were grown in a humidified environment at 37°C and 5% CO_2_. The cultured media were changed every 48 h. Cells used in the experiments were from passages 4 to 6. HAECs were cultured in the presence of HG (D-glucose, 40 mM) and PA (palmitate, a saturated fatty acid that has been associated with cellular toxicity, 0.1 mM) with different doses of GSC extracts and metformin for 48 h. In all experiments involving treatment with PA-BSA, BSA served as control. Chemical inhibitors or activators, CCCP (20 *μ*M), bafilomycin A1 (5 nM), Mdivi-1 (2 *μ*M), and compound C (5 *μ*M), were added as indicated in Results. For adenoviral infection, Ad-GFP-LC3 (Hanbio Biotechnology, China) was used following the manufacturer's instructions.

### 2.3. Cell Proliferation Assay

HAECs (5 × 10^3^ cells/well) were seeded in 96-well plates. After adherence, the cells were incubated in ECM with or without HG/PA and GSC extracts. After 48 h of incubation, 10 *μ*L of CCK-8 (Dojindo, Japan) solution and 90 *μ*L of ECM were added to each well, and the cells were further incubated in the CO_2_ incubator at 37°C for 2 or 3 h. The absorbance was measured at 450 nm using a Synergy H1 Automatic Microplate Reader (BioTek, USA).

### 2.4. SA-*β*-Gal Staining

SA-*β*-gal activity was determined in HAECs as per the protocol. Briefly, cells were fixed with SA-*β*-gal staining fixative for 15 min, washed three times with DPBS, and incubated with SA-*β*-gal staining working solution. After 16-24 h of being incubated at 37°C without CO_2_, images were taken using an AE 2000-type inverted phase contrast microscope and imaging system (Motic, China).

### 2.5. Immunofluorescence

After 48 h of treatment, HAECs were fixed with 4% paraformaldehyde for 15 min, followed by three washes with DPBS. Cells were permeabilized with 0.1% Triton-X 100 for 10 min and blocked with 5% BSA for 1 h at room temperature. After blocking, the cells were incubated with *γ*-H2AX (1 : 500, Cat No. 9718, CST) or Parkin (1 : 100, Cat No. 66674-1-lg, Proteintech) antibody overnight at 4°C. Following incubation with Alexa Flour 488 secondary antibody for 1 h at room temperature, the cells were incubated with DAPI stain for 5 min to stain the nucleus. Next, cells were observed under a FV1000 confocal microscope (Olympus, Japan).

For mitophagy detection, GFP-LC3-transfected cells were incubated with 100 nM MitoTracker™ Red CMXRos (Cat No. M7512, Thermo Fisher Scientific) that was diluted in serum-free media at 37°C for 30 min. Afterwards, they were analyzed by confocal microscopy.

### 2.6. mtROS and Mitochondrial Membrane Potential(MMP)

The production of mtROS was measured by MitoSOX Red mitochondrial superoxide indicator (Cat No. M36008, Invitrogen). HAECs were incubated with 5 *μ*M MitoSOX reagent working solution for 10 min at 37°C in the dark. Then, cells were washed gently three times with warm buffer. Red fluorescence was measured at the excitation/emission maxima of approximately 510/580 nm using a confocal microscope.

MMPs were assessed using the Mitochondrial Membrane Potential Assay Kit with JC-1 (Cat No. C2006, Beyotime). HAECs were seeded and treated for 48 h. After experimentation, cells were treated with JC-1 staining working solution in a dark environment for 20 min and washed twice with cold staining buffer. Green fluorescence reflected the monomeric form of JC-1, and red fluorescence reflected the aggregated form of JC-1. The cells were monitored using a confocal microscope.

### 2.7. Western Blot

HAECs were lysed with RIPA buffer, protease inhibitor, and phosphatase inhibitor. The protein concentrations were measured with the BCA method. Equivalent amounts of total protein were separated by SDS-PAGE and transferred to PVDF membranes. Nonspecific binding was blocked with 5% nonfat milk (dissolved in PBS) for 1 h and then incubated overnight at 4°C with antibodies: p16 (1 : 1000, Cat No. ab51243, Abcam), p21 (1 : 1000, Cat No. ab109199, Abcam), LC3B (1 : 2000, Cat No. ab192890, Abcam), p62 (1 : 2000, Cat No. ab56416, Abcam), PINK1(1 : 1000, Cat No. 23274-1-AP, Proteintech), Parkin, AMPK*α* (1 : 2000, Cat No. ab32047, Abcam), p-AMPK*α* (1 : 1000, Cat No. 2535, CST), mTOR (1 : 1000, Cat No. ab32028, Abcam), p-mTOR (1 : 1000, Cat No. 5536, CST), p70S6K (1 : 1000, Cat No. 2708, CST), p-p70S6K (1 : 1000, Cat No. 9208, CST), and *β*-actin (1 : 2000, Cat No. ab16039, Abcam). All membranes were washed with TBST three times and incubated for 1 h at room temperature with a secondary antibody (1 : 3000, ZSGB-BIO, China) that had been dissolved in TBST. Finally, the membranes were washed three times with TBST. The blots were then developed using an ECL detection kit (Cat No. WBKLS0100, Millipore, USA). The developed blots were subjected to grayscale analysis by Image Lab 6.0 and normalized with an internal control.

### 2.8. Short Hairpin RNA (shRNA) Transfection

Coding sequences for shRNA targeted to prkaa1 or for scrambled shRNA were inserted into the plenti-U6-egfp-puro plasmid, which also encoded with enhanced green fluorescent protein (EGFP). The shRNA target sequences were 5′-GCAGAAGTATGTAGAGCAATC-3′. The constructed plenti-prkaa1 shRNA and plenti-Scramble shRNA were cotransfected with 293T cells with a lentiviral backbone plasmid at a mass ratio 3 : 5. The 293T cells were in a 100 mm culture dish and were treated with replaced fresh complete medium (DMEM + 10% FBS) after 8 h, and the supernatant was collected at 48 h and 72 h, respectively. After being filtered, concentrated, and resuspended, the virus particles were collected at -80°C. The titers of LV-prkaa1 shRNA and LV-Scramble shRNA were 2.5 × 10^8^ TU and 1.0 × 10^8^ TU, respectively. HAECs were transfected with each shRNA and incubated at 37°C. At 8 h posttransfection, the media were refreshed, and HAECs were treated for 48 h with HG/PA, GSC extracts, or metformin.

### 2.9. Statistical Analysis

All results were expressed as the means ± SE. Comparisons between two groups were calculated using Student's *t*-test. For comparisons involving more than two groups, one-way analysis of variance (ANOVA) with a post hoc Bonferroni multiple comparison test was used to assess the difference. All statistical analyses were performed with GraphPad Prism version 6.0 software. *P* < 0.05 was considered statistically significant.

## 3. Results

### 3.1. GSC Extracts Prevent HG/PA-Induced Cellular Senescence in HAECs

**Figure 2:**
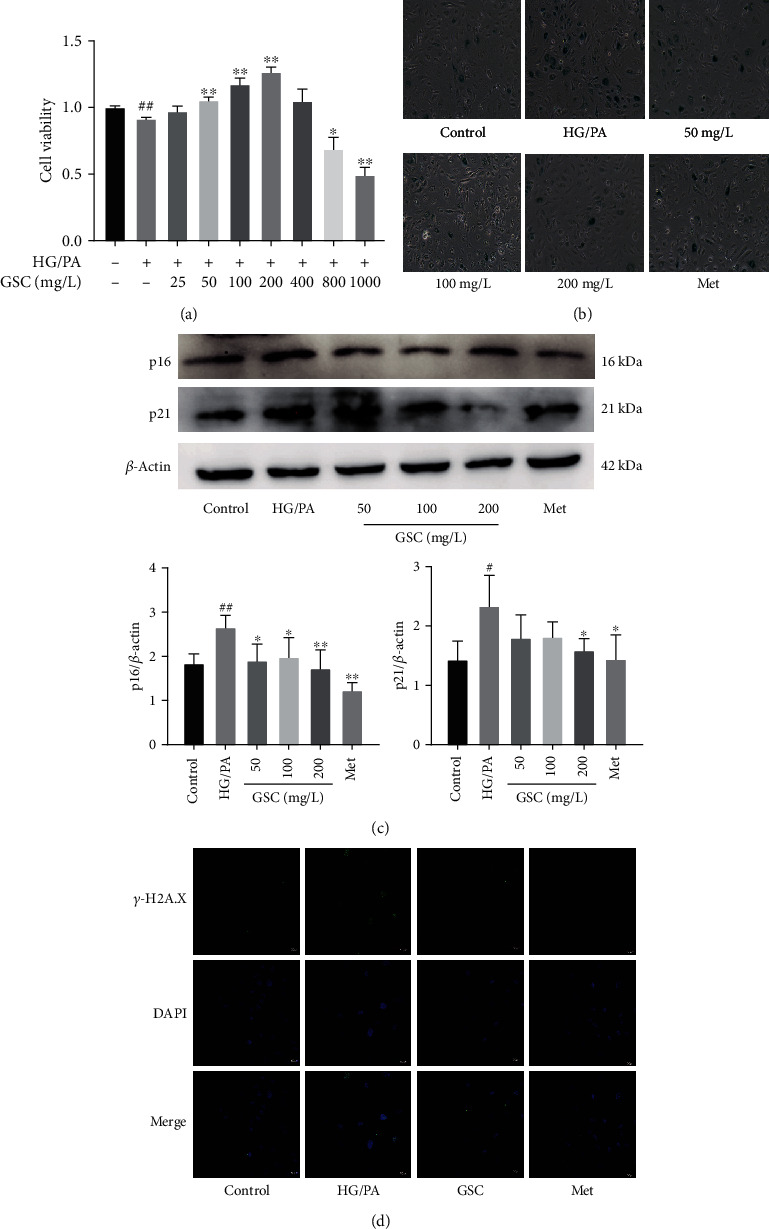
GSC extracts prevent HG/PA-induced cellular senescence in HAECs. HAECs were treated with varying concentrations of GSC extracts in the presence of 40 mM glucose and 0.1 mM palmitate for 48 h. (a) Cell viability as determined by CCK-8 assay. (b) SA-*β*-gal were detected in HAECs. (c) Western blot analysis of the expression of p16 and p21 in HAECs. (d) Confocal images to show *γ*-H2AX focus expression in HAECs after 48 h. ^#^*P* < 0.05 and ^##^*P* < 0.01, compared with the control group; ^∗^*P* < 0.05 and ^∗∗^*P* < 0.01, compared with the model (HG/PA) group.

PA is the main type of saturated fatty acid in plasma and is substantially elevated in subjects with diabetes who have metabolic syndrome. To mimic the diabetic microenvironment in vitro, HAECs were treated with HG and PA for 48 h. CCK-8 was applied for the determination of the cell proliferation viability. It was observed that subjecting to 0.1 mM PA and 40 mM glucose decreased the viability of HAECs by approximately 10%, whereas coincubation with GSC extracts gradually improved cell viability in a dose-dependent manner under 200 mg/L concentration (Figure 2(a)). Furthermore, HG/PA-treated HAECs became enlarged and flattened and were characterized by increased SA-*β*-gal activity. On the other hand, cotreatment with GSC extracts led to decreased numbers of SA-*β*-gal blue-stained cells and staining was nearly absent at the treatment with a concentration of 200 mg/L (Figure 2(b)). Increased cyclin-dependent kinase inhibitors, including p16 and p21, as well as DNA damage are other hallmarks for cellular senescence. Our results showed that increased p16 and p21 levels as well as *γ*-H2AX focus formation in HG/PA-treated HAECs. In contrast, the expressions of p16, p21, and *γ*-H2AX foci were attenuated by GSC extracts after 48 h of cotreatment (Figures 2(c) and 2(d)). Together, these findings suggest the cytoprotective and antisenescent effects of GSC extracts against HG/PA-stressed HAECs.

### 3.2. GSC Extracts Regulate Mitochondrial Function in Senescent HAECs

**Figure 3:**
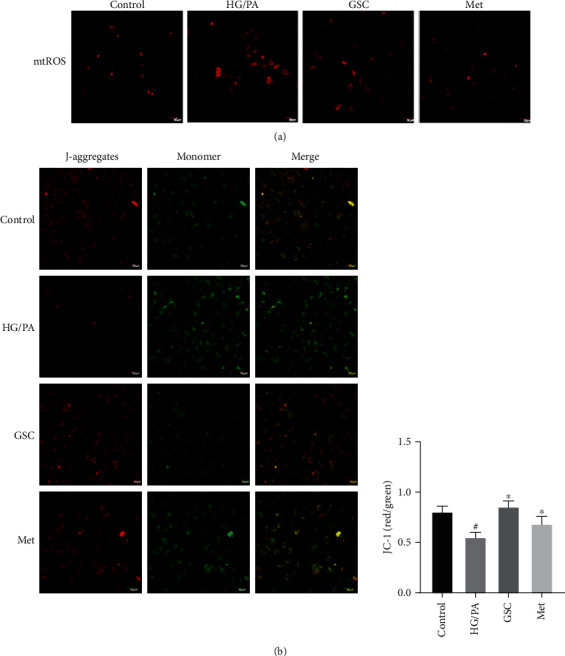
GSC extracts regulate mitochondrial function in senescent HAECs. (a) Confocal microscope examination of MitoSOX fluorescence staining. (b) Mitochondrial membrane potentials were determined by a JC-1 probe.

It is known that increased mtROS induces DNA damage associated with cellular senescence. To investigate whether the production of mtROS could be affected in senescent HAECs under HG/PA-stressed conditions, MitoSOX fluorescence staining was used to detect mtROS. The results showed that increased mtROS was significantly inhibited by GSC extracts and metformin (Figure 3(a)). Injured mitochondria are the main source of mtROS, which is characterized by MMP changes. As shown in the results, a decline of MMP level was detected by a JC-1 probe in the HG/PA group (green fluorescence and the green-to-red ratio were significantly increased compared with the control group), whereas GSC extracts and metformin increased the level of MMP (Figure 3(b)).

### 3.3. GSC Extracts Restore Impaired Mitophagy in HG/PA-Induced Cellular Senescence in HAECs

**Figure 4:**
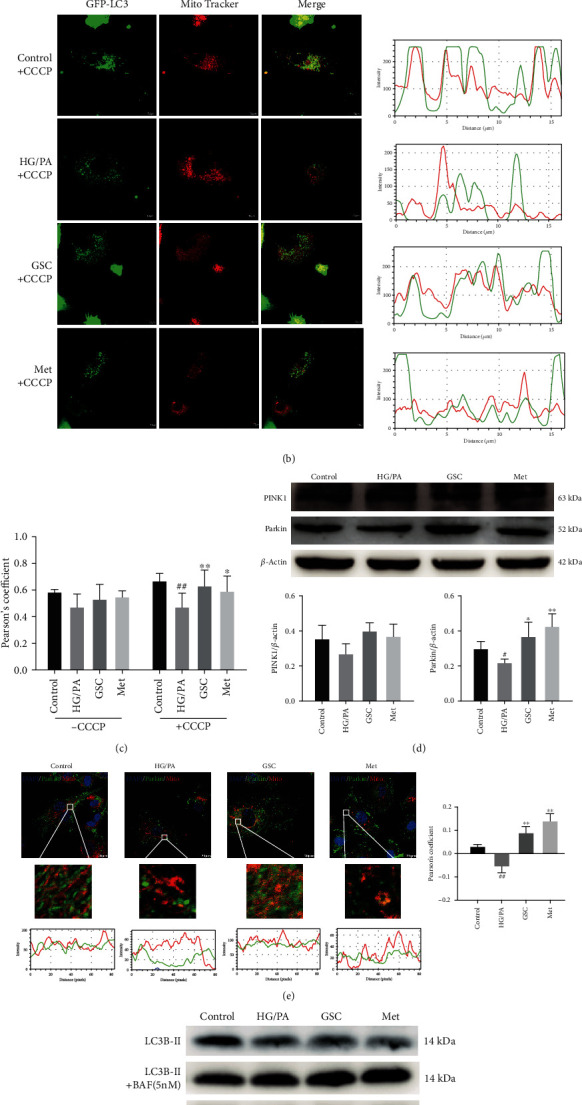
GSC extracts restore impaired autophagy and mitophagy in HG/PA-induced cellular senescence in HAECs. (a) Representative images of GFP-LC3 expressing HAECs stained with MitoTracker Red after 48 h of treatment. Square scan data of fluorescence intensity in the corresponding images to show the degree of colocalization between LC3 and mitochondria. (b) HAECs were treated with HG/PA for 48 h followed by treatment with 20 *μ*M CCCP for 4 h. The image shows the colocalization of GFP-LC3 with mitochondria (MitoTracker Red) after CCCP treatment. (c) Pearson correlation coefficient shows the degree of colocalization between LC3 and mitochondria (MitoTracker Red). (d) Western blot analysis of PINK1 and Parkin protein expression in cultured HAECs. (e) Image shows the colocalization of Parkin and mitochondria in HAECs. (f, g) Western blot analysis of LC3B-II and p62 in HAECs followed by treatment with or without bafilomycin A1 for 4 h. ^#^*P* < 0.05 and ^##^*P* < 0.01, compared with the control group; ^∗^*P* < 0.05 and ^∗∗^*P* < 0.01, compared with the model (HG/PA) group.

The production of mtROS is associated with damaged mitochondria. Damaged mitochondria are mainly eliminated by selective autophagy, and dysfunctional mitochondria are engulfed into autophagosomes and lysosomal compartments for degradation (a process termed mitophagy). In this study, we investigated whether treatment with GSC extracts led to any changes in mitophagy via colocalization of autophagy marker GFP-LC3 (green) and the MitoTracker probe (red). After treatment with GSC extracts, there was little colocalization (yellow dots) of autophagosome marker LC3 and mitochondria in senescent HAECs treated with HG/PA or GSC extracts. To verify the defective mitophagy in senescent HAECs, we found that treatment with CCCP (a well-known inducer of mitophagy) can reduce the colocalization of LC3 and mitochondria in HG/PA-treated HAECs, whereas the colocalization was highly induced after cotreatment with 200 mg/L GSC extracts; this indicates that damaged mitophagy was restored by GSC extracts (Figures 4(a)–4(c)).

PINK1-Parkin pathway is the main mechanism of mitophagy, which can recruit autophagosome to the damaged mitochondria [26–28]. Therefore, we determined the expression of PINK1 and Parkin protein in HAECs. Compared with normal conditions, HG/PA-stressed conditions reduced the expression of PINK and Parkin protein. Immunostaining showed that HG/PA not only decreased Parkin level but also inhibited Parkin recruitment to mitochondria. In contrast to this, the expression of both PINK1 and Parkin protein increased under HG/PA-stressed conditions when cotreated with GSC extracts, and the level of Parkin had an upward trend with statistical significance. Moreover, treatment with GSC extracts also promoted Parkin colocalization to mitochondria in HG/PA-stressed HAECs (Figures 4(d) and 4(e)).

Next, we further investigated the formation of autophagosomes and autophagic flux. When autophagosomes were formed, the cytosolic protein LC3-I was converted to LC3-II by enzymatic hydrolysis, and the elevation of LC3-II represented the initiation of autophagy. Bafilomycin A1 could prevent the formation of the autophagosome-lysosome complex, which helped us to determine whether the increase of autophagosomes is solely due to the induction of autophagy itself or simply due to impairment in autophagy turnover. As shown in Figures 4(f) and 4(g), HG/PA-stress decreased LC3B-II/*β*-actin expression, and cotreatment with GSC extracts could dramatically increase the expression of LC3B-II/*β*-actin in the presence of bafilomycin A1. In addition, the ubiquitin-binding receptor protein p62 accumulated on the depolarized mitochondria and facilitated the recruitment of damaged mitochondria to lysosomes by binding to LC3, which thereby completed the selective clearance of mitochondria. The level of p62 is inversely proportional to autophagy flux. Immunoblotting analysis showed that the expression of p62 protein in HAECs treated with GSC extracts and HG/PA was significantly reduced, indicating that GSC extracts could manifest the induction of autophagosome formation without impairing autophagy turnover.

Overall, these data strongly suggest that by regulating the PINK1/Parkin pathway, GSC extracts might not only restore mitophagy but also induce autophagic activity, which is partly impaired by chronic HG/PA stimulation.

### 3.4. Mitophagy Inhibition Eliminates the Effect of GSC Extracts on Preventing HG/PA-Induced Cellular Senescence

**Figure 5:**
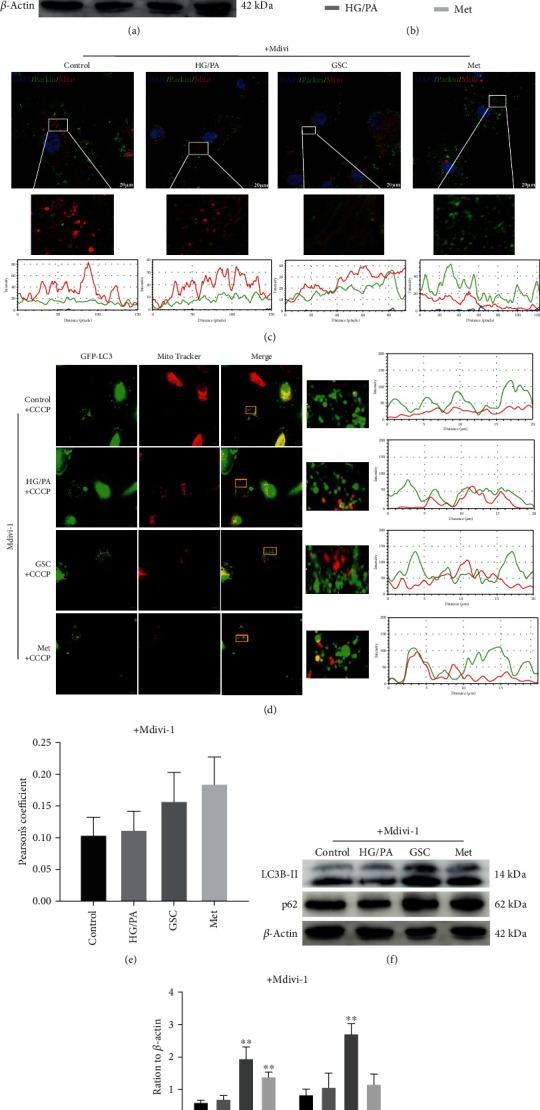
Mdivi-1 pretreatment for 1 h abolished the induction of mitophagy by GSC extracts. (a, b) Western blot analysis of the expression of PINK1 and Parkin protein in cultured HAECs after pretreatment with 2 *μ*M Mdivi-1. (c) Image shows the colocalization of Parkin protein and mitochondria in HAECs after pretreatment with 2 *μ*M Mdivi-1. (d) HAECs were treated with Mdivi-1 for 48 h followed by treatment with 20 *μ*M CCCP for 4 h. Image shows the colocalization of GFP-LC3 with mitochondria after treatment with CCCP. (e) Pearson correlation coefficient shows the degree of colocalization between LC3 and mitochondria. (f, g) Western blot analysis of LC3B-II and p62 for 48 h in cultured HAECs after pretreatment with 2 *μ*M Mdivi-1. ^#^*P* < 0.05 and ^##^*P* < 0.01, compared with the control group; ^∗^*P* < 0.05 and ^∗∗^*P* < 0.01, compared with the model (HG/PA) group.

To further determine whether impaired mitophagy is involved in the cytoprotective effect of GSC extracts in HG/PA-induced cellular senescence, the specific mitochondrial division/mitophagy inhibitor Mdivi-1 was applied in following study. Pretreatment with Mdivi-1 (2 *μ*M) abolished the induction of mitophagy by GSC extracts. As shown in Figures 5(a) and 5(c), Mdivi-1 averted the Parkin protein increase and the recruitment induced by GSC extracts. Moreover, the additive effect of Mdivi-1 was also evident on GFP-LC3, which showed a decrease in colocalization with mitochondria, to indicate impaired mitophagy. Treatment with CCCP could not induce colocalization of GFP-LC3 with mitochondria after treatment with GSC extracts and Mdivi-1 in HAECs (Figures 5(d) and 5(e)). The expressions of LC3B-II and p62 increased in HG/PA-stressed HAECs after cotreatment with GSC extracts and Mdivi-1, indicating that there might be a blockage of autophagic flux (Figures 5(f) and 5(g)).

**Figure 6:**
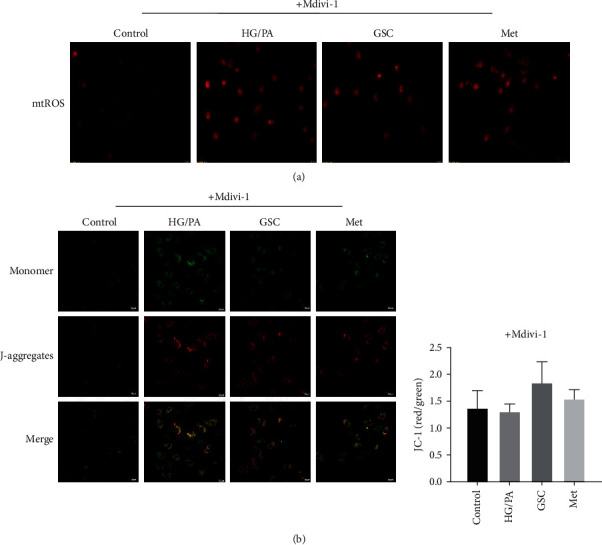
Pretreatment with Mdivi-1 abolished the effect of GSC extracts on mitochondria function. (a) Confocal microscope examination of MitoSOX fluorescence staining. (b) Mitochondrial membrane potentials were determined by a JC-1 probe.

We next investigated whether the effect of GSC extracts on mitochondrial function could be abolished by Mdivi-1. Under HG/PA-stressed conditions, we found that GSC extracts and metformin cannot inhibit the production of mtROS and increase the level of MMP when cotreatment with Mdivi-1 (Figure 6).

**Figure 7:**
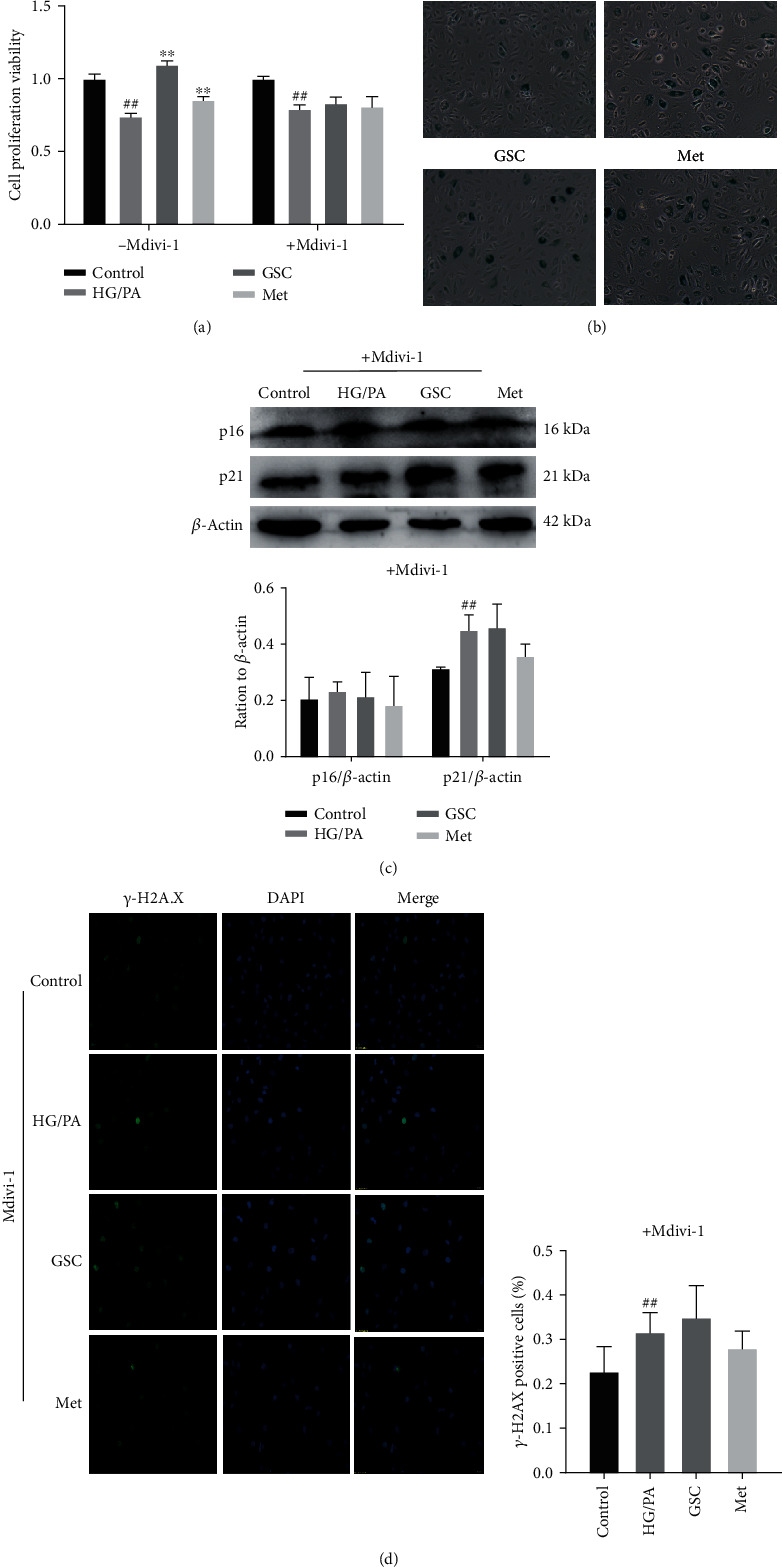
Pretreatment with Mdivi-1 reversed the effect of GSC extracts against HG/PA-induced cellular senescence in HAECs. (a) Cell viability as determined by CCK-8 assay. (b) SA-*β*-gal were detected in HAECs. (c) Western blot analysis of p16 and p21 expression in HAECs. (d) Confocal images show *γ*-H2AX focus expression in HAECs. ^#^*P* < 0.05 and ^##^*P* < 0.01, compared with the control group; ^∗^*P* < 0.05 and ^∗∗^*P* < 0.01, compared with the model (HG/PA) group.

As a result, pretreatment with Mdivi-1 reversed the effect of GSC extracts against HG/PA-induced cellular senescence in HAECs. As shown in Figure 7, cotreatment with GSC extracts and Mdivi-1 could not decrease the levels of SA-*β*-gal, p16, p21, and *γ*-H2AX. These results confirmed that the restoration of damaged mitophagy by GSC extracts is an important prerequisite for inhibition of HG/PA-induced cellular senescence.

### 3.5. GSC Extracts Regulate Mitophagy through AMPK Signaling Pathway

**Figure 8:**
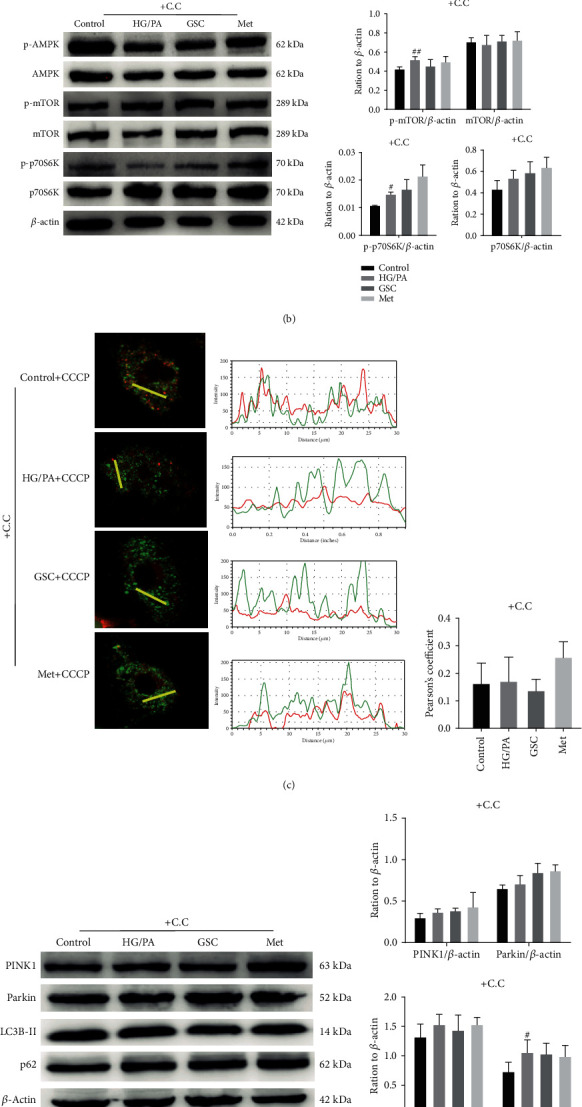
GSC extracts regulate mitophagy through the AMPK signaling pathway; pretreatment with compound C (5 *μ*M) for 1 h abolished GSC extract-induced mitophagy in HAECs. (a) AMPK signaling pathway expression in HG/PA-induced senescent HAECs. (b) Western blot analysis of AMPK, p-AMPK, mTOR, p-mTOR, p70S6K, and p-p70S6K expression in cultured HAECs after pretreatment with compound C. (c) HAECs were treated with compound C for 48 h followed by treatment with CCCP (20 *μ*M) for 4 h. Image shows the colocalization of GFP-LC3 with mitochondria after treatment with CCCP. Pearson's coefficient shows the degree of colocalization between LC3 and mitochondria. (d) Western blot analysis of PINK1, Parkin, LC3B-II, and p62 expression in cultured HAECs with compound C for 48 h. (e) Image shows the colocalization of Parkin and mitochondria in HAECs after pretreatment with compound C. ^#^*P* < 0.05 and ^##^*P* < 0.01, compared with the control group; ^∗^*P* < 0.05 and ^∗∗^*P* < 0.01, compared with the model (HG/PA) group.

It is already known that the AMPK signaling pathway plays an essential role in regulating both mitophagy and cellular senescence. To further clarify the mechanism driving GSC extract-mediated mitophagy and senescence, the expression of the AMPK pathway was checked in HAECs. As shown in Figure 8(a), significant decreases in the protein levels of AMPK and p-AMPK were observed under HG/PA-stressed conditions, and an inverse activation of its downstream targets p-mTOR and p-p70S6K was also observed. Treatment with GSC extracts and metformin upregulated AMPK protein and its phosphorylation level, and it downregulated the phosphorylation level of mTOR and p70S6K, demonstrating an activation of the AMPK signaling pathway.

**Figure 9:**
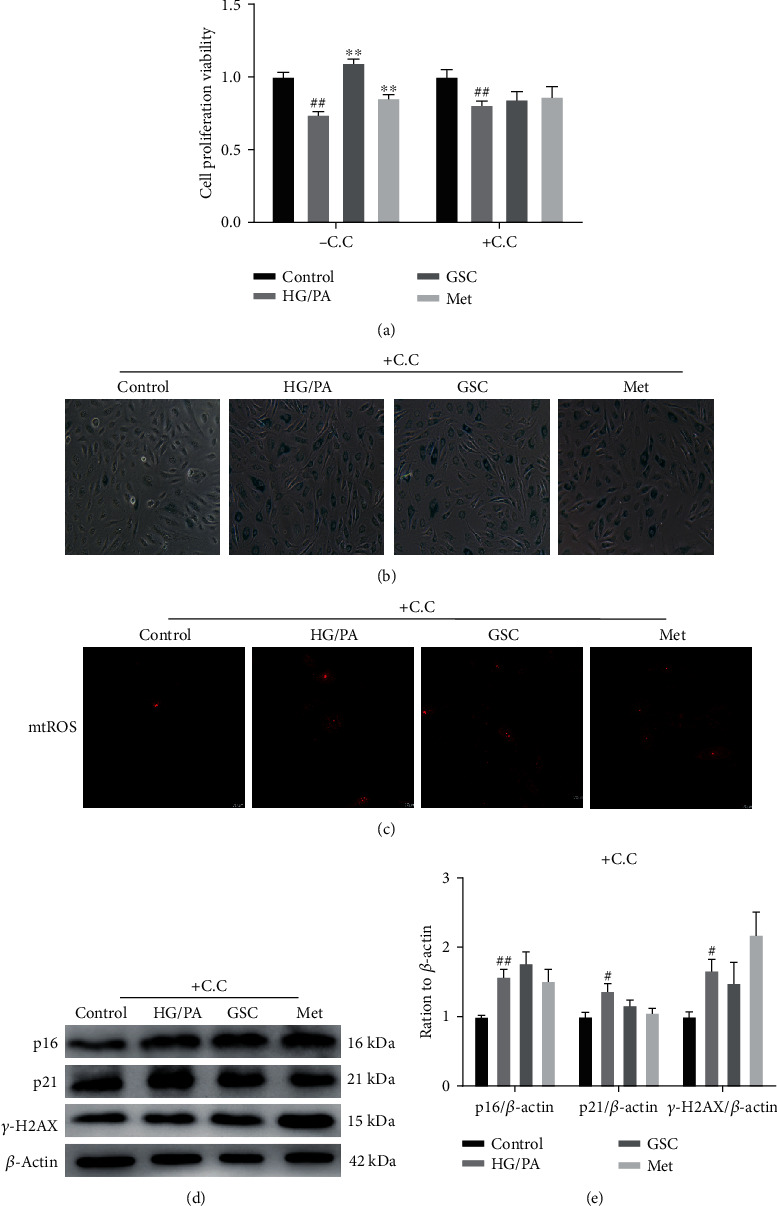
Pretreatment with compound C reversed the effect of GSC extracts against HG/PA-induced cellular senescence in HAECs. (a) Cell viability as determined by CCK-8 assay. (b) SA-*β*-gal were detected in HAECs. (c) Confocal images to show mtROS production in HAECs. (d, e) Western blot analysis of p16, p21, and *γ*-H2AX expression in HAECs. ^#^*P* < 0.05 and ^##^*P* < 0.01, compared with the control group; ^∗^*P* < 0.05 and ^∗∗^*P* < 0.01, compared with the model (HG/PA) group.

To confirm the role of GSC extracts in activating the AMPK signaling pathway, a chemical AMPK inhibitor was used in the study. As shown in Figure 8(b), GSC extract-induced upregulation of AMPK phosphorylation and downregulation of mTOR and p70S6K phosphorylation were reversed in the presence of compound C. With the inhibition of the AMPK signaling pathway, treatment with GSC extracts and metformin could not increase the colocalization of autophagosomes and mitochondria, and it had no positive effect on Parkin, LC3-II, and p62 in HAECs treated with HG/PA, indicating a failed activation of mitophagy (Figures 8(c)–8(e)). Moreover, GSC extracts lost their ability to inhibit HG/PA-induced senescence in HAECs when exposed to compound C (Figure 9).

**Figure 10:**
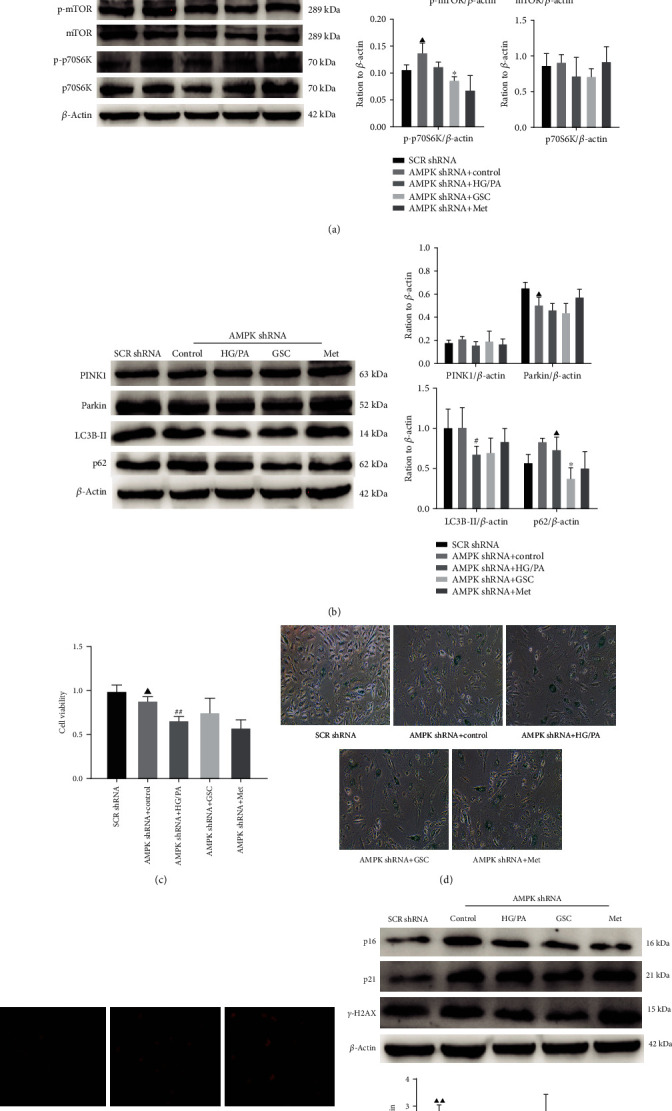
AMPK inhibition by shRNA reversed the effect of GSC extracts against HG/PA-induced cellular senescence in HAECs. HAECs were transfected with AMPK shRNA or SCR shRNA for 8 h and then treated with HG/PA, GSC extracts, or metformin for 48 h. (a) Western blot analysis of AMPK, p-AMPK, mTOR, p-mTOR, p70S6K, and p-p70S6K expression in transfected HAECs. (b) Western blot analysis of PINK1, Parkin, LC3B-II, and p62 expression in transfected HAECs. (c) Cell viability as determined by CCK-8 assay. (d) SA-*β*-gal were detected in HAECs. (e) Immunofluorescence images to show mtROS production in HAECs. (f) Western blot analysis of p16 and p21 expression in HAECs. ^▲^*P* < 0.05 and ^▲▲^*P* < 0.01, compared with the SCR shRNA group. ^#^*P* < 0.05 and ^##^*P* < 0.01, compared with the control group; ^∗^*P* < 0.05 and ^∗∗^*P* < 0.01, compared with the model (HG/PA) group.

To further confirm the specific involvement of AMPK of GSC extracts, we genetically inhibited AMPK with shRNA lentiviral particles. Compared with SCR shRNA transfection (negative control group), AMPK*α*1 shRNA dramatically downregulated the level of AMPK protein and its phosphorylation, while it simultaneously upregulated the phosphorylation levels of its downstream targets mTOR and p70S6K; this induced a significant reduction in Parkin protein expression and increased accumulation of p62 in HAECs. As expected, there was decreased cell proliferation and increased number of SA-*β*-gal blue-stained cells, mtROS production, and expression of p16, p21, and *γ*-H2AX proteins in AMPK*α*1 shRNA-transfected HAECs (Figure 10). These results suggested that AMPK*α*1, a catalytic subunit of AMPK, plays an important role in mitophagy and senescence in HAECs. As shown in Figure 10(a), in HG/PA-stressed AMPK*α*1 shRNA-transfected HAECs, treatment with GSC extracts had no effect on AMPK expression and downregulated the levels of mTOR and p70S6K phosphorylation; however, the degree of downregulation was obviously lower than what is shown in Figure 8. Western blot analysis showed that GSC extracts failed to restore the expression of Parkin and LC3-II but still reduced the expression of p62 in AMPK*α*1 shRNA-transfected HAECs, indicating that AMPK*α*1 inhibition attenuated the promotion of mitophagy by GSC extracts without affecting autophagic flow (Figure 10(b)). Moreover, AMPK*α*1 shRNA interference almost abolished the effects of GSC extracts on cell proliferation, SA-*β*-gal-positive staining, mtROS production, and p21 expression (Figures 10(c)–10(f)). Together, the results demonstrated that GSC extract-mediated protection against HG/PA-induced cellular senescence in HAECs via mitophagy activation was partially dependent on the AMPK*α*1 signaling pathway.

## 4. Discussion

This study is the first to demonstrate that GSC extracts can attenuate HG/PA-induced cellular senescence in HAECs by inducing mitophagy via activating the AMPK signaling pathway. Endothelial cellular dysfunction is identified to be a major hallmark of vascular injury, which leads to the development of vascular complications in diabetes [29, 30]. Previous studies have demonstrated that increased vascular endothelial cellular senescence contributes to endothelial dysfunction [31], and identification reagents or mechanisms for protecting endothelial cells from chronic metabolic stress-induced cellular senescence have become the focus of attention in the treatment of diabetes mellitus. A number of natural and synthetic compounds have been investigated for their antisenescence and antiaging potential in cellular and animal models as well as humans [32, 33], including metformin, rapamycin, and resveratrol. However, some synthetic molecules may cause significant adverse effects, and the mechanism of some natural compounds is still unclear. GSC extracts from Chinese herbs have been used to treat cardiovascular diseases, such as hypertension and coronary heart disease, for thousands of years. In this study, we treated primary HAECs with HG/PA simulating the pathological stress of poorly controlled diabetes and demonstrated the novel role of GSC extracts in HG/PA-induced cellular senescence in HAECs. Treatment with GSC extracts reduced the hallmarks of cellular senescence in SA-*β*-gal, p16, and p21 but increased cell proliferation in a dose-dependent manner. Additionally, DNA damage marker was also decreased in HAECs cotreated with GSC extracts.

Previous studies have reported that HG or PA usually induces oxidative stress, thereby damaging the cell by increasing the production of intracellular ROS. Excessive ROS is mainly generated by dysfunctional mitochondria and leads to oxidative damage of membranes and macromolecules (such as DNA and proteins), which in turn accelerates cellular senescence [34]. In the resent study, we found that G/PA promoted mtROS production and decreased MMP in senescent HAECs. This was in line with studies [35] that dysfunctional mitochondria and excessive mtROS generation might be responsible for the effects of HG/PA on senescent HAECs. Previous studies have demonstrated GSC extracts could improve senescence of vascular endothelial cells by influencing the RAAS system, NO synthesis, and oxidative stress. In the final analysis, these are related to inhibit mtROS production. In HG/PA conditions, we found that GSC extracts could also alleviate mtROS overproduction and mitochondrial damage in senescent HAECs; however, the mechanism is unknown.

Mitophagy is activated by mitochondrial membrane depolarization, and it is then followed by stabilization of PINK1 on the mitochondrial outer membrane, recruitment of E3 ubiquitin ligase Parkin from the cytosol, and amplification of the initiation signals of mitophagy [10]. Mitophagy might clear damaged mitochondria, thereby inhibiting the excessive production of mtROS. Whether PINK1-Parkin pathway-mediated mitophagy is involved in chronic metabolic stress induced cellular senescence is not fully understood. A recent study found that PINK1 and Parkin were upregulated in vessel wall of obese and diabetic mice and were activated by treatment with lower dose PA in cultured endothelial cells, which can prevent ROS production by promoting mitophagy [36]. In addition, under HG conditions, a significant increase in the association of Parkin with damaged mitochondria as well as the colocalization of mitochondrial protein COXIV with autophagosome marker LC3B-II and the lysosomal membrane protein LAMP2A was observed in a rat Müller cell line [37]. However, contrary to these studies, some reports revealed inhibition of mitophagy under a certain degree of HG/PA-stressed conditions. Song et al. have found that metabolic stressed ob/ob mice and HG/PA-treated HepG2 cells showed inhibition of mitophagy by disturbing translocation of Parkin to mitochondria [38]. Another study has reported a reduction in mitophagy-associated proteins (Parkin, LC3B, and beclin 1) in HG/PA-stressed RAECs [39]. Consistent with these reports, we observed a suppression of mitophagy in HG/PA-stressed HAECs. Our results showed that there was a limited colocalization of mitochondria with LC3 and Parkin in HAECs under HG/PA-stressed conditions. This was accompanied by an increase in the expression of LC3 and p62 proteins. Together, it could be assumed that HG/PA treatment leads to suppression of mitophagy and turnover of autophagosomes in HAECs.

Impaired mitophagy is responsible for accumulation of damaged mitochondria and cellular senescence. Both PINK1 and Parkin knockout mice show elevated ROS levels and mitochondrial dysfunction [40, 41]. In contrast, activation of mitophagy by Parkin overexpression has been shown to delay senescence in Drosophila and reduce oxidative stress [42]. In the present study, we have shown that GSC extracts prompted mitophagy and increased turnover of autophagosomes in HG/PA-induced HAECs. Our results revealed that GSC extracts induced colocalization of mitochondria with LC3 and Parkin, which was accompanied by increased LC3B-II, PINK1, and Parkin and decreased p62 expression. Furthermore, inhibition of GSC extract-mediated mitophagy by Mdivi-1 abolished the mitochondrial protection and antisenescent potential of GSC extracts. This further reinforced our hypothesis that GSC extract-mediated mitophagy exerts antisenescent effects on HG/PA-stressed HAECs.

In subsequent studies, molecular mechanism involved in the GSC extract-mediated induction of mitophagy was analyzed. AMPK widely presents in eukaryotic cells and is a highly conserved serine/threonine kinase that cooperates with downstream molecules to play an important role in the regulation of mitophagy [16, 17, 43]. Studies have shown that AMPK could directly form a stable complex with ULK1, an autophagic promoter, and activate ULK1 translocation to mitochondria by phosphorylating the Ser555, Thr574, and Ser637 sites of ULK1 and initiating mitophagy to remove oxidative stress-damaged mitochondria [44, 45]. mTOR is the downstream target of AMPK that inhibits autophagy by phosphorylation of ULK1 and preventing AMPK-ULK1 interactions. Activation of mTORC1 also leads to defective mitophagy (such as impaired or senescent mitochondria accumulation, altered PINK1 expression, and abnormal translocation of Parkin to damaged mitochondria), and mTORC1 inhibitors reverse these changes, which strongly suggests that mTOR regulates mitophagy through the PINK1/Parkin signaling pathway [46]. Based on these observations, we further investigated the role of the AMPK signaling pathway in GSC extract-induced cytoprotection. Our results showed that GSC extracts activated the AMPK pathway, as evidenced by the increased AMPK and p-AMPK (Thr172) and decreased p-mTOR and p-p70S6K expressions in senescent HAECs. Moreover, pharmacological and genetic inhibition of AMPK abolished GSC extract-induced mitophagy and subsequently aggravated senescence in HG/PA-stressed HAECs. We also noticed that the changes of total AMPK protein were inconsistent with some reports [47, 48]. However, we have repeatedly verified that the protein levels of total AMPK and p-AMPK have indeed changed, indicating that HG/PA simultaneously inhibits the expression and activity of AMPK, and GSC extracts can simultaneously increase the expression and activity of AMPK. Changes in AMPK activity may be caused by changes in AMPK expression. As for the reasons for inconsistency with some reports, we believe that it might be related to factors such as cell type, drug type, and drug intervention time.

**Figure 11:**
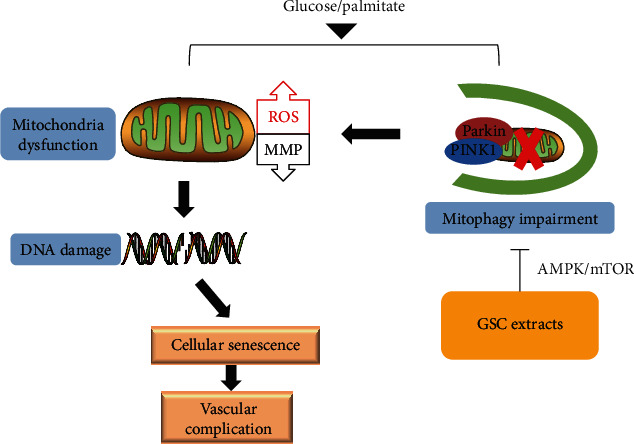
The critical role of GSC extracts in antisenescent actions by inducing mitophagy via the AMPK signaling pathway in HG/PA-stressed HAECs.

In summary, our senescent HAEC model of poorly controlled type 2 diabetes, using chronic exposure to glucose and palmitate, revealed that these nutrients impaired mitophagy and increased oxidative stress, resulting in cellular senescence. All results provide evidence of a critical role of GSC extracts in antisenescent actions by inducing mitophagy via the AMPK signaling pathway in HG/PA-stressed HAECs (Figure 11). To our knowledge, this study provides new insights into our understanding of potential treatment with GSC extracts and indicates what might be a promising candidate for preventing endothelial senescence as well as diabetic vascular complications.

However, the present study has some limitations. First, autophagy is a double-edged sword in diseases, so dynamic changes of mitophagy needed to be observed in subsequent studies, and further investigation of the effects of GSC extracts on endothelial aging in vivo is also clearly needed. Such experiments will help us to evaluate whether GSC extracts modulate mitophagy via the AMPK signaling pathway in vivo.

## Data Availability

The data used to support the findings of this study are included within the article.

## Supplementary Materials

Supplementary Materials Supplemental Figure 1 and Table 1: representative chromatogram and contents of main compounds in Ginseng. Supplemental Figure 2 and Table 2: representative chromatogram and contents of main compounds in Sanqi. Supplemental Figure 3 and Table 3: representative chromatogram and contents of main compounds in Chuanxiong. Supplemental Figures 4 and 5: representative chromatogram of ferulic acid, notoginsenoside R1, ginsenoside Rg1, ginsenoside Re, and ginsenoside Rb1 in GSC extracts. Supplemental Table 4: contents of main compounds in GSC extracts.
