# Supplementary material for: Ginseng-Sanqi-Chuanxiong (GSC) Extracts Ameliorate Diabetes-Induced Endothelial Cell Senescence through Regulating Mitophagy via the AMPK Pathway
Source: Oxid Med Cell Longev. 2020 Sep 7;2020:7151946. doi: 10.1155/2020/7151946 (PMC7495226; doi:10.1155/2020/7151946)
Supplement: Supplementary Materials — Supplemental Figure 1 and Table 1: representative chromatogram and contents of main compounds in Ginseng. Supplemental Figure 2 and Table 2: representative chromatogram and contents of main compounds in Sanqi. Supplemental Figure 3 and Table 3: representative chromatogram and contents of main compounds in Chuanxiong. Supplemental Figures 4 and 5: representative chromatogram of ferulic acid, notoginsenoside R1, ginsenoside Rg1, ginsenoside Re, and ginsenoside Rb1 in GSC extracts. Supplemental Table 4: contents of main compounds in GSC extracts. [file 7151946.f1.docx]

**Main Compounds of Ginseng-Sanqi-Chuanxiong(GSC) Extracts Analysis**

1. Determination of major compounds of ginseng, Sanqi and Chuanxiong

In order to ensure the repeatability of experiment, the single herbs and GSC extracts used in this study were tested for the content of main compounds using high-performance liquid chromatography (HPLC). According to the pharmacopoeia standards, we have determined the content of ginsenoside Rg1, ginsenoside Re and ginsenoside Rb1 in Ginseng (origin: Jilin, batch number: YL-302-1403-001) [1]; the content of ginsenoside Rg1, ginsenoside Rb1 and notoginsenoside R1 in Sanqi (origin: Yunnan, batch number: YL-201-1403-001) [2]; the content of ferulic acid in Chuanxiong (origin: Sichuan, batch number: YL-018-1403-001) [3], as shown in Figure 1-3 and Table 1-3, respectively.


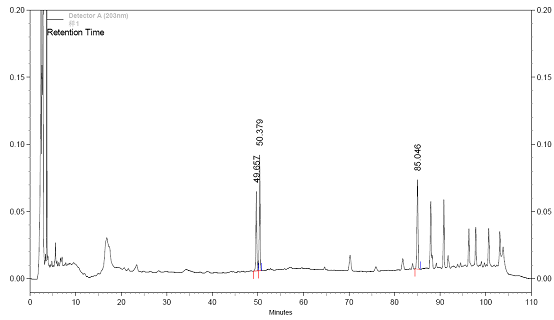


Fig.1 Representative chromatogram of ginsenoside Rg1, ginsenoside Re and ginsenoside Rb1 in Ginseng

Table 1 Contents of main compounds in Ginseng

| Compoounds | Contents（%） |
| --- | --- |
| ginsenoside Rg1 | 0.23 |
| ginsenoside Re | 0.28 |
| ginsenoside Rb1 | 0.37 |


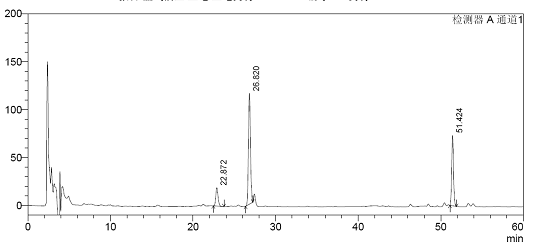


Fig.2 Representative chromatogram of ginsenoside Rg1, ginsenoside Rb1 and notoginsenoside R1 in Sanqi

Table 2 Contents of main compounds in Sanqi

| Compoounds | Contents（%） |
| --- | --- |
| notoginsenoside R1 | 1.09 |
| ginsenoside Rg1 | 5.54 |
| ginsenoside Rb1 | 3.54 |


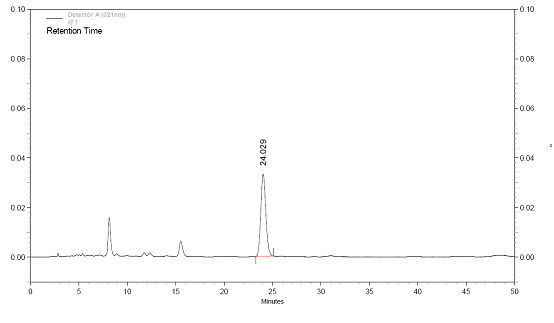
Fig.3 Representative chromatogram of ferulic acid in Chuanxiong

Table 3 Contents of main compounds in Chuanxiong

| Compoounds | Contents（%） |
| --- | --- |
| ferulic acid | 0.22 |

2. Determination of major compounds in GSC extracts

According to the pharmacopoeia, major compounds of Ferulic acid, Notoginsenoside R1, Ginsenoside Rg1, Ginsenoside Re and Ginsenoside Rb1 in GSC extracts was determined, as shown in Figure 4-5 and Table 4.


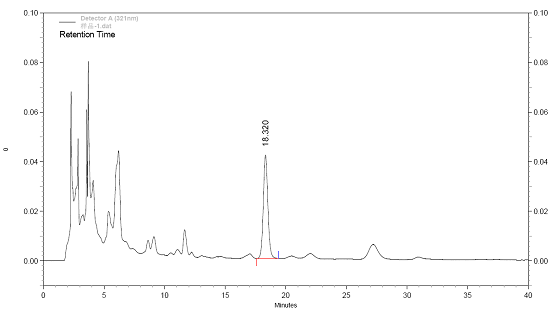


Fig.4 Representative chromatogram of ferulic acid in GSC extracts


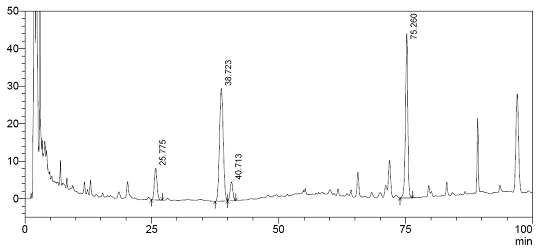


Fig.5 Representative chromatogram of notoginsenoside R1, ginsenoside Rg1, ginsenoside Re and ginsenoside Rb1 in GSC extracts

Table 4 Contents of main compounds in GSC extracts

| Compoounds | Contents（%） |
| --- | --- |
| ferulic acid | 1.00 |
| notoginsenoside R1  ginsenoside Rg1  ginsenoside Re  ginsenoside Rb1 | 8.45  55.84  6.47  44.57 |

**Reference**

[1] National Pharmacopoeia Commission. Pharmacopoeia of the People's Republic of China [M]. Beijing: China Medical Science and Technology Press, 2015: 8.

[2] National Pharmacopoeia Commission. Pharmacopoeia of the People's Republic of China [M]. Beijing: China Medical Science and Technology Press, 2015: 11.

[3] National Pharmacopoeia Commission. Pharmacopoeia of the People's Republic of China [M]. Beijing: China Medical Science and Technology Press, 2015: 40.
